# A validated high-fidelity synthetic model for internal mammary vessel preparation in autologous breast reconstruction

**DOI:** 10.1016/j.jpra.2026.09.014

**Published:** 2026-09-16

**Authors:** Iulia Marinescu, Adam F. Roche, Caoimhin O’Conghaile, Claire Condron, Omar Quidwai, Stephanie Bollard, Barry O’Sullivan, Jamie Martin Smith, Jack Woods, Colin Morrison, Adrian McArdle, Anton Fries, Roisin Dolan

**Affiliations:** a Department of Plastic and Reconstructive Surgery, St. Vincent’s University Hospital, Dublin, Ireland; b UCD School of Medicine & Medical Sciences, University College Dublin, Ireland; c RCSI SIM Centre for Simulation Education and Research, RCSI University of Medicine and Health Sciences, Dublin, Ireland; d Department of Plastic and Reconstructive Surgery, Beaumont Hospital, Dublin, Ireland; e Department of Surgery, Division of Plastic, Reconstructive & Aesthetic Surgery, Temerty Faculty of Medicine, University of Toronto, Toronto, Canada; f Division of Plastic Surgery, UT Health San Antonio, San Antonio, TX, United States

**Keywords:** Internal mammary vessels, Breast reconstruction, Microsurgery, Surgical simulation, Technical skills assessment, Objective structured assessment of technical skills

## Abstract

**Aims:**

The precise and safe preparation of internal mammary vessels (IMVs) is a critical step in microvascular autologous breast reconstruction. These vessels lie within a confined, dynamic space, requiring refined dexterity and careful tissue handling. Opportunities for structured training remain limited. This study aimed to develop and validate a high-fidelity synthetic model for IMV preparation and assess its educational value.

**Materials & Methods:**

A synthetic post-mastectomy chest model was designed in collaboration with specialist technicians with expertise in simulation design and implementation, with iterative input from consultant microsurgeons. Five novice and five expert surgeons participated. Face and content validity were assessed by independent expert evaluation. Construct validity was examined using a task-specific checklist and the Objective Structured Assessment of Technical Skills (OSATS), scored by two blinded assessors. Novice confidence was assessed pre- and post-training.

**Results:**

Expert evaluation demonstrated good face validity, with high ratings for overall realism and anatomical fidelity (4.5/5), vessel dissection (3.25/5) and teaching suitability and utility for higher surgical training (4.25/5). Construct validity was supported by higher expert performance (OSATS: 23.5 vs 18.9; checklist: 10.0 vs 9.4), and faster task completion time (13.8 vs 15.8 min). Inter-rater reliability was moderate to strong (ICC 0.575–0.820). Novice confidence improved significantly following training (mean increase 2.6, t = 6.50, p = 0.003).

**Conclusions:**

This realistically responsive training model demonstrates strong face, content and construct validity. It provides a practical and objective platform for training and assessing internal mammary vessel preparation and supports safer skill acquisition before progression to the operating theatre.

## Introduction

Autologous free tissue transfer is a well-established approach to post-mastectomy reconstruction and requires advanced microsurgical expertise.^1^ The internal mammary vessels (IMVs) have been the most commonly employed recipient vessels since the 1990s and successful anastomosis requires meticulous preparation and adequate exposure of these vessels.^2^, ^3^, ^4^, ^5^ Preparation of the IMVs involves resection of the overlying 3rd or 4th costal cartilage and its periosteal sleeve in a confined space. This step can be technically demanding, particularly in irradiated patients and carries the risk of vessel injury and pneumothorax.^6^ Despite its technical importance, opportunities for trainees to practice IMV preparation in a structured environment, outside the operating theatre, are limited.^7^

Simulation is increasingly incorporated into surgical training, offering a controlled and psychologically safe environment for deliberate practice and feedback.^8^, ^9^, ^10^ Effective surgical simulation models should demonstrate anatomical fidelity, be feasible for repeated use and be supported by objective assessment frameworks capable of distinguishing between levels of expertise.^11^ Whilst cadaveric and animal models provide highest realism, their use is constrained by ethical, logistical and financial considerations. In lieu of this, synthetic simulation models have become a practical alternative.^12^ Current education guidance recommends validation using Messick’s unified validity framework which requires multiple sources of evidence including expert appraisal and demonstration of construct validity.^13^

To address the absence of a validated procedural simulator for IMV exposure, we developed a synthetic model for IMV dissection that recreates the key anatomical layers and enables learners to practise the technical steps required for safe exposure. The aim of this study was to evaluate the validity and educational value of the synthetic training model for plastic surgery trainees.

## Materials and methods

This study was designed and reported in accordance with the methodological recommendations outlined for validation research in surgical education.^14^

Validity was conceptualised using Messick’s unified framework, incorporating multiple sources of evidence, including content, response process, internal structure, relations to other variables (construct validity), and consequences.^15^ Ethical approval was obtained from via application 202,411,006, and all participants provided informed consent prior to participation in this research.

### Simulation model

Design-based research (DBR) is commonly applied to improve educational practices, technologies, and designs.^16^ Practitioner-education researcher collaboration is a key feature of DBR where iterative micro-cycles of design and implementation allow evaluation and incremental improvements. The four steps of the DBR methodology, as described by Reeves (2006) were followed through the problem analysis and simulation solution design.^17^ A spiral approach combining both design and prototyping-in-stages and incorporating both clinical and simulation concepts allows us to align the model to specific learning outcomes requirements.^18^

The simulator was designed to provide both visual and haptic feedback and consists of a reusable plastic torso covered with silicone ‘skin’. Two 8.5 × 5 cm chest slots hold expendable cartridges containing a segment of the 3rd rib constructed from expandable foam, with overlying perichondrium simulated using micropore tape. Pectoral and intercostal muscles, adipose tissue, and internal mammary vessels are made from silicone. Vessels are filled with simulated blood to provide realistic feedback during dissection. The model underwent iterative refinement over a three-month period, informed by repeated feedback from experienced microsurgeons, until consensus was reached that all key procedural steps could be reliably performed. The procedural steps demonstrated on the model are outlined in Fig. 1.

**Fig. 1:**
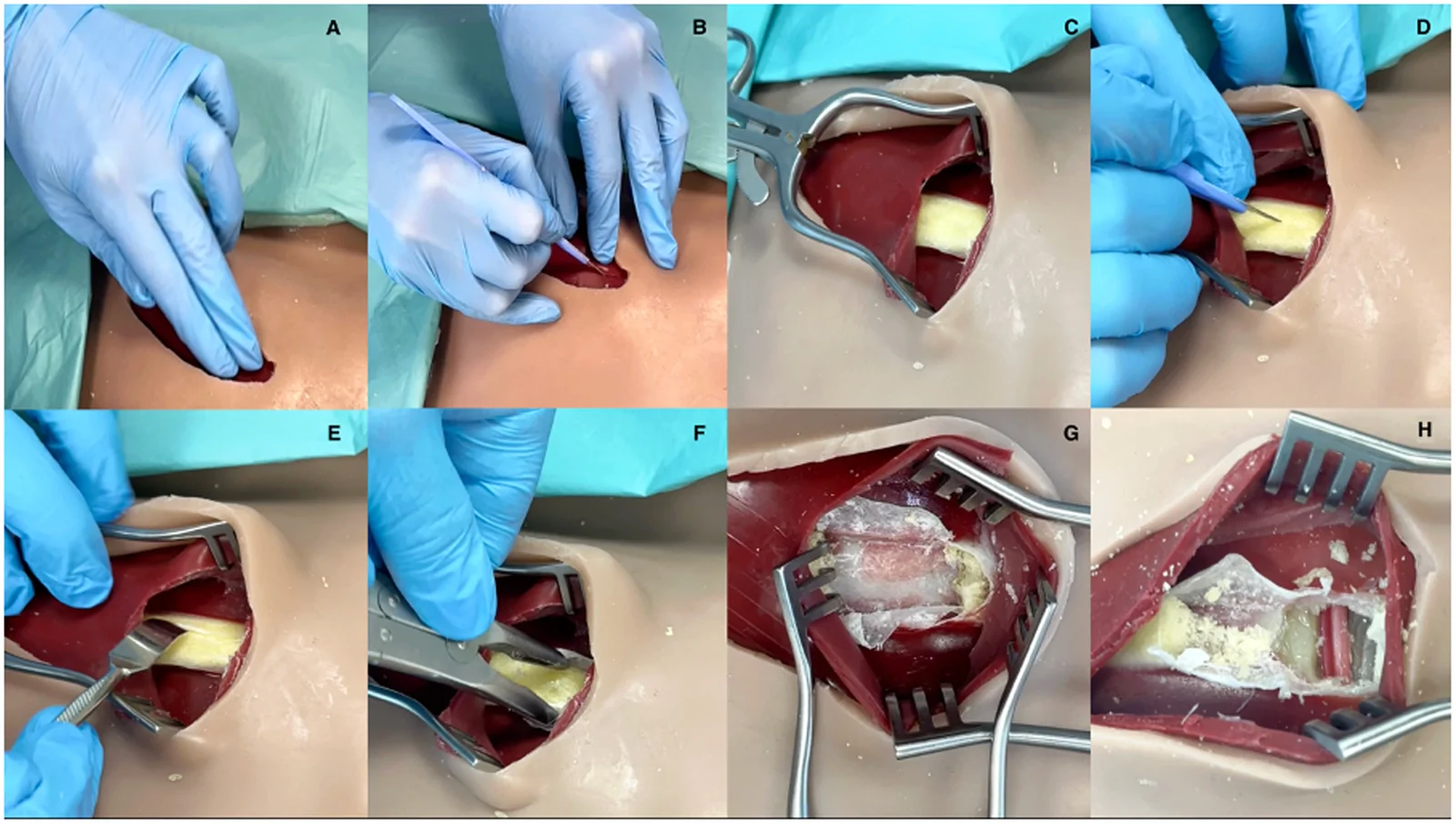
Stepwise preparation of internal mammary recipient vessels on the synthetic chest model: A, identifying the 3rd rib; B, pectoralis muscle incision; C, exposure of the 3rd rib; D-E, dissection of the perichondrium; F, excision of the costal cartilage; G, opening the posterior perichondrium; H, exposure of the internal mammary vessels.

### Participants

Five novice and five expert surgeons were recruited. The novice group comprised four senior house officers (SHOs) and one junior registrar in plastic surgery, each with 2–3 years of postgraduate clinical experience and no prior experience in IMV dissection. The expert group consisted of four consultant microsurgeons and one senior specialist registrar (year 7 of 8), all with independent experience in IMV preparation.

### Simulation protocol and response process

Response process validity was addressed by using a standardisation of the simulation environment, including scripted participant instructions, a uniform teaching intervention and trained assessors. All novice participants attended a teaching session delivered by a senior trainee, including a step-by-step instructional video of IMV preparation. Prior to task performance, all participants viewed a demonstration of the procedure on the simulation model.

Participants were then instructed to independently perform isolation of the internal mammary artery and vein(s) in preparation for autologous breast reconstruction. Standardised instruments were provided, and assistance from a study author (IM) was available if required. Participants did not wear magnification loupes during the simulated procedure. Performances were video- recorded anonymously and stored on a secure platform for subsequent blinded assessment.

### Outcome measures

#### Validity assessment

**Content validity** was assessed through expert evaluation of anatomical realism and educational utility. Expert participants completed a seven-item questionnaire adapted from a previously validated tool,^18^ using a 5-point Likert scale. Median scores and ranges were calculated, and responses were compared to the neutral midpoint using one-sample Wilcoxon signed-rank tests.

**Construct validity** was evaluated by comparing performance between novice and expert groups. Procedural performance was assessed using the Objective Structured Assessment of Technical Skills (OSATS), comprising six domains scored on a 5-point Likert scale (maximum score of 35).^19^ A task-specific checklist was also employed, consisting of nine procedural steps scored as complete or incomplete, alongside a global competency rating (0–2). Assessment tools were developed by the senior authors based on established procedural descriptions.^20^ All performances were independently scored by two blinded expert assessors (AF and AMA). Scores were reported as mean ± standard deviation (SD). Group comparisons were performed using Welch t-tests and Wilcoxon rank-sum tests as appropriate. To assess internal structure, inter-rater reliability was evaluated using a two-way mixed-effects intraclass correlation coefficient (ICC(3,k)) in accordance with the Shrout and Fleiss framework.^21^

#### Impact

Trainee confidence was assessed using pre- and post-simulation questionnaires completed by the novice cohort. Confidence was measured on a 10-point Likert scale (1 = minimal confidence, 10 = maximal confidence. Differences between pre- to post-training scores were analysed using a paired t-test.

All statistical analyses were conducted using R (R Core Team, 2024) within the RStudio integrated development environment.^22^

## Results

### Content validity

Participant demographics are summarised in Table 1. Median (range) scores across the eight content validity items were: skin realism 4 (4–5), soft tissue realism 4 (4–5), third rib realism 5 (4–5), perichondrium realism 5 (4–5), IMV dissection realism 4 (2–4), overall simulator realism 4 (4–5), suitability for teaching IMV isolation 5 (3–5), and usefulness for higher surgical training 5 (3–5). Seven of the eight items were rated significantly above the neutral midpoint (p < 0.05), including skin, soft tissue, bony and perichondral anatomy, overall realism, and perceived educational utility. Only IMV dissection realism did not differ significantly from the midpoint (p = 0.21) (Table 2).

**Table 1:** Participant demographics.

	Trainee	Expert
Number of participants	5	5
Gender (n)		
Male	2	3
Female	3	2
Median age (range)	29 (26–31)	42 (33–56)
Hand dominance (n)		
Left	0	1
Right	4	4
Ambidextrous	1	0
Median times observed/assisted (range)	0 (0–5)	100 (30–300)
Median times performed (range)	0 (0–0)	100 (10–300)

**Table 2:** Content validity questionnaire responses (1–5) and one-sample Wilcoxon signed-rank test results assessing whether ratings were significantly greater than the midpoint score of 3.

Item	Mean	SD	Median	Range	p value
Realistic representation of skin	4.4	0.55	4	4–5	0.03
Realistic representation of soft tissue layers	4.4	0.55	4	4–5	0.03
Realistic representation of the 3rd rib	4.8	0.45	5	4–5	0.02
Realistic representation of perichondrium	4.8	0.45	5	4–5	0.02
Realistic representation of IMV dissection	3.4	0.89	4	2–4	0.21
Overall realism of simulator	4.4	0.55	4	4–5	0.03
Suitable to teach IMV isolation steps	4.4	0.89	5	3–5	0.04
Useful for training HSTs	4.4	0.89	5	3–5	0.04

IMV, internal mammary vessels; HSTs, higher specialty trainees; SD, standard deviation.

### Construct validity

#### OSATS and task-specific checklist

Experts outperformed novices across all performance measures. Mean task-specific checklist scores were 9.4 ± 1.08 for novices and 10.0 ± 1.41 for experts. Mean OSATS scores were 18.9 ± 2.65 for novices and 23.5 ± 3.42 for experts. Experts also completed the task more quickly (13.8 ± 4.97 min vs 15.8 ± 6.87 min) (Table 3).

**Table 3:** Performance descriptive statistics of the novice and expert groups.

Group	Task-specific checklist mean score (SD)	OSATS mean score (SD)	Time to task completion mean (SD)
Novice	9.4 (1.08)	18.9 (2.65)	15.8 (6.87)
Expert	10 (1.41)	23.5 (3.42)	13.8 (4.97)

SD, standard deviation.

Group comparisons demonstrated a consistent trend favouring expert performance. Differences in OSATS scores showed a trend towards higher scores among experts (t = –2.19, p = 0.061; 95% CI: –9.48 to 0.28). Differences in task completion time (t = 0.53, p = 0.614; 95% CI: –6.90 to 10.90) and checklist scores (W = 16, p = 0.515) were not statistically significant (Table 4).

**Table 4:** Statistical comparison of OSATS score, procedure time, and checklist performance between novice and expert groups.

Outcome (Test)	Test Statistic	p-value	95% CI (Lower)	95% CI (Upper)
OSATS (Welch t-test)	−2.1905	0.0614	−9.4812	0.2812
Time (Welch t-test)	0.5274	0.6136	−6.8959	10.8959
Checklist (Wilcoxon rank-sum)	16	0.515	NA	NA

OSATS, objective structured assessment of technical skills; CI, confidence interval.

#### Inter-rater reliability

Inter-rater reliability was acceptable for both assessment tools. The task-specific checklist demonstrated moderate reliability (ICC(3,k = 0.575), while the OSATS scale demonstrated strong reliability (ICC(3,k) of 0.820) (Table 5).

**Table 5:** Inter-rater reliability based on the average scores of two independent expert assessors.

Scale	ICC_3k
Task-specific checklist	0.575
OSATS	0.820

ICC, intraclass correlation coefficient; OSATS, objective structured assessment of technical skills.

### Pre- and post-test evaluation

Trainee confidence increased following simulation training, from a mean of 3.0 ± 2.12 pre-training to 5.6 ± 1.52 post-training. This represented a statistically significant increase in confidence of 2.6 points (95% CI 1.49 to 3.71, t = 6.50, p = 0.003. The magnitude of change was large (Cohen’s dz = 2.91). These findings should be interpreted with caution given the sample size. (Table 6)

**Table 6:** Student’s t-test results comparing trainee confidence before and after training on the simulation model.

Confidence	Mean (SD)	t-value	p-value	95% CI	p-value	Effect size (dz)
Pre-training	3.0 (2.1)	6.5	0.0029	1.489 to 3.711	0.0029	2.91
Post-training	5.6 (1.5)					

SD, standard deviation; CI, confidence interval.

## Discussion

Preparation of the IMVs can represent a technically demanding component of autologous breast reconstruction, requiring precise dissection within a confined anatomical space. Despite its importance, opportunities for structured training are limited, with most learning occurring opportunistically in the operating theatre. This creates a gap between theoretical knowledge and technical execution, particularly for early-stage trainees.

This study addresses the gap through the development and validation of a high-fidelity, procedure-specific synthetic model. The simulator demonstrated evidence of validity across multiple domains. Expert users rated the model highly for anatomical realism and educational utility, supporting content validity. Construct validity was supported by consistent differences in performance between expert and novice groups across global rating scales, task-specific checklist scores, and task completion time, indicating that the model can discriminate between levels of surgical experience. Inter-rater reliability was acceptable to strong, further supporting the robustness of the assessment framework.

These findings are consistent with the broader surgical education literature which demonstrates that simulation-based training facilitates technical skill acquisition, accelerates learning curves, and improves trainee confidence in a safe, reproducible environment.^23^, ^24^, ^25^, ^26^ This simulator was designed as a procedure-specific platform targeting the key technical steps and anatomical challenges unique to IMV preparation, as targeted simulation is more likely to translate into meaningful surgical competence. Training using real tissue, whether animal or cadaveric, certainly offers unique advantages in terms of realism. The present model does not intend to substitute these established training practices, but it is designed as an accessible adjunct, particularly for repeated practice or when opportunities to practice on real tissue are limited or reserved for higher stages of training.

A critical consideration in simulation-based education is the need for robust validation to ensure that training tools are aligned with defined learning objectives and appropriate to the target trainee population.^27^ The IMV simulator was developed through iterative expert input to ensure both anatomical relevance and procedural realism. High ratings for skin, soft tissue, rib, and perichondral realism reflect successful replication of the operative environment. Greater variability in ratings for IMV dissection likely reflects the inherent complexity of replicating microvascular structures in synthetic materials, but overall scores remained favourable. Importantly, the inclusion of both visual and haptic feedback enables trainees to develop an appreciation of tissue planes and force application, which are critical to avoidance of complication such as IMV injury and pneumothorax.

In the context of IMV identification, both rib-sparing and rib-sacrificing techniques are common practice based on surgeon preference. Rib-sacrificing approaches are generally considered more reproducible for early stage training, whereas rib-sparing techniques demand greater technical precision.^28^^,^^29^ The current model is optimised for training in the rib-sacrificing method, aligning with the needs of less experienced trainees and appropriately targets foundational skill acquisition. Adaption to incorporate rib-sparing techniques may be possible in future iterations, however fewer technical steps are challenged in this approach, potentially limiting its educational value at early stages of training.

The ability of the simulator to discriminate levels of experience is particularly relevant in microsurgical education, where objective assessment of technical skill remains challenging.^30^ Although some between-group comparisons did not reach statistical significance, likely reflecting the small sample size, the direction and magnitude of the observed effects were consistent with theoretical expectations. Emphasis on effect sizes and confidence intervals, rather than p-values alone, supports the interpretation that the model possesses construct validity. Strong inter-rater reliability confirms consistency of assessment across expert evaluators, reinforcing the reliability of the evaluation framework. Thus, use of objective performance metrics helps mitigate the limitations of purely subjective rating scales in simulation‑based training, and our approach is consistent with validation strategies used for porcine and robotic internal mammary artery simulators.^5^^,^^31^

In addition to objective performance metrics, use of a simulator was associated with a significant improvement in trainee confidence. Whilst confidence is not a surrogate for competence, simulation-based practice using benchtop models represents an important early outcome that may facilitate engagement with supervised operative training.^32^^,^^33^ These findings align with existing literature demonstrating favourable effects on learner reaction, satisfaction, and perceived readiness for clinical performance.^34^

Several limitations should be acknowledged. The small sample size limits generalisability and statistical power, and findings should be interpreted with caution. The model does not currently replicate perforators or small branching vessels, which represent critical challenges encountered in real-life IMV dissection.^35^^,^^28^ Incorporation of these features in future iterations of the model may enhance procedural realism. In addition, this study does not assess transfer validity, and further work is required to determine whether skills acquired in simulation translate into improved operative performance.

Future research should focus on evaluating the impact of simulation-based training on clinical performance and exploring integration of this model within structured microsurgical training curricula. This study supports the use of validated synthetic simulation as a core adjunct in microsurgical training, enabling progression from simulation to supervised clinical practice.

## Conclusion

This study supports the validity of a novel, procedure-specific simulation model for IMV preparation. Its ability to reliably assess performance, discriminate between levels of expertise, and improve trainee confidence supports its use as a structured adjunct to microsurgical training, facilitating progression to supervised clinical practice.

## Funding statement

This study was funded by the HSE Academic Medical Internship research bursary.

This work was presented at the Sylvester O’Halloran Perioperative Symposium, University of Limerick School of Medicine, Ireland, on 27 February 2026 and received the Best Poster Award in the General Poster Session.

## Ethical approval

Granted by the RCSI REC through application number 202,411,006.

## Declaration of competing interest

The authors declare no conflict of interest.
